# Climate Change Impacts Assessment Using Crop Simulation Model Intercomparison Approach in Northern Indo-Gangetic Basin of Bangladesh

**DOI:** 10.3390/ijerph192315829

**Published:** 2022-11-28

**Authors:** Md Rafique Ahasan Chawdhery, Murtuza Al-Mueed, Md Abdul Wazed, Shah-Al Emran, Md Abeed Hossain Chowdhury, Sk Ghulam Hussain

**Affiliations:** 1 Department of Agroecology and Crop Production, Faculty of Agrobiology, Food and Natural Resources, Czech University of Life Sciences Prague, Kamýcká 129, Suchdol, 16500 Prague, Czech Republic; 2 Ministry of Public Administration, Dhaka 1000, Bangladesh; 3 Department of Chemical and Materials Engineering, University of Auckland, Private Bag 92019, Auckland 1142, New Zealand; 4 Department of Crop Sciences, University of Illinois at Urbana-Champaign, Urbana, IL 61801, USA; 5 Bangladesh Agricultural Research Council, Crop Zoning Project, Farmgate, Dhaka 1000, Bangladesh; 6 International Maize and Wheat Improvement Center (CIIMMYT), Gulshan-1, Dhaka 1212, Bangladesh

**Keywords:** APSIM and DSSAT crop simulation models, climate change impact, cropping system, Indo-Gangetic Basin (IGB), integrated approach

## Abstract

The climate change impacts of South Asia (SA) are inextricably linked with increased monsoon variability and a clearly deteriorating trend with more frequent deficit monsoons. One of the most climate-vulnerable nations in the eastern and central Indo-Gangetic Basin is Bangladesh. There have been numerous studies on the effects of climate change in Bangladesh; however, most of them tended to just look at a small fraction of the impact elements or were climatic projections without accounting for the effects on agriculture. Additionally, simulation studies using the CERES-Rice and CERES-Wheat models were conducted for rice and wheat to evaluate the effects of climate change on Bangladeshi agriculture. However, up to now, Bangladesh has not implemented farming system ideas by integrating cropping systems with other income-generating activities. This study was conducted as part of the Indo-Gangetic Basin (IGB) regional evaluations using the protocols and integrated assessment processes of the Agricultural Model Intercomparison and Improvement Project (AgMIP). It was also done to calibrate crop models (APSIM and DSSAT) using rice and wheat. To assist policymakers in creating national and regional plans for anticipated future agricultural systems, our work on the integrated evaluation of climate change impacts on agricultural systems produced realistic predictions. The outcome of this research prescribes a holistic assessment of climate change on future production systems by including all the relevant enterprises in the agriculture sector. The findings of the study suggested two major strategies to minimize the yield and increase the profitability in a rice–wheat cropping system. Using a short-term HYV (High Yielding Variety) of rice can shift the sowing time of wheat by 7 days in advance compared to the traditional sowing days of mid-November. In addition, increasing the irrigation amount by 50 mm for wheat showed a better yield by 1.5–32.2% in different scenarios. These climate change adaptation measures could increase the per capita income by as high as 3.6% on the farm level.

## 1. Introduction

Climate change impacts are increasingly visible in South Asia (SA), with greater variability of the monsoon, noticeably, a declining trend with more frequent deficit monsoons [1]. Bangladesh is one of the most climate-vulnerable countries in the central and eastern Indo-Gangetic Basin. The rapidly growing population of the country puts tremendous pressure on its scarce natural resources. The country is vulnerable to many climatic hazards, including frequent floods, droughts, cyclones, and storm surges that damage life, property, and agricultural production [2]. Climate change could make it more difficult than it already is to accelerate agricultural production to meet the growing demands in countries in the region and more specifically for Bangladesh. The above-mentioned types of disasters make the problems more complicated. In the foreseeable future, Bangladesh is likely to be one of the most vulnerable countries in the world in the event of climate change [3]. Global warming due to the increase in greenhouse gas concentrations in the earth’s atmosphere and the consequent sea level rise is adding fuel to the fire. Almost every sector of socio-economic life in Bangladesh is likely to be affected by climate change. The 6th Assessment Report of the Intergovernmental Panel on Climate Change (IPCC) has recently reported that “Warming of the climate system is unequivocal, and since the 1950s, many of the observed changes are unprecedented over decades to millennia. The atmosphere and ocean have warmed, the amounts of snow and ice have diminished, sea level has risen, and the concentrations of greenhouse gases have increased” [4]. Climatic factors (e.g., temperature, precipitation, wind, etc.) regulate agricultural activities and security in production [5,6]. In developing countries, such as Bangladesh, poor households mostly live in rural areas and are largely dependent on the agricultural production system, and therefore, climate change has a major impact on their livelihood and food security [7,8].

There have been several studies of climate change impacts in Bangladesh, but they have provided climate projections without quantifying agricultural impacts or tended to examine just a subset of the impact factors. For example, some studies of climate change impacts on Bangladesh rice have focused on temperature and carbon dioxide effects [9,10]. Additionally, simulation studies were carried out for rice and wheat using the APSIM-Oryza and APSIM-Wheat models and CERES-Rice and CERES-Wheat for DSSAT to assess the impact of climate change on Bangladesh agriculture [11,12]. The Agricultural Production Systems Simulator (APSIM) is a software package that facilitates sub-modules combined to simulate agricultural systems [13]. The APSIM simulator has several modules categorized as Plant-Soil-Atmosphere and crop management operations. The model can simulate crop growth stage developments, soil processes, and crop management options. The APSIM model requires an input dataset of weather, soil, and crop management development on a daily basis to run the simulation outputs. The DSSAT cropping model is a computer-based crop growth application that can simulate growth, development and yield of crop growing under defined management over time [14,15]. The DSSAT model requires databases of daily basis weather data, soil, crop management conditions and cultivar-specific genotypes to simulate and compare results with observed estimates. Moreover, a number of investigations have been made in the Indo-Gangetic area to achieve sustainable land use. For instance, a conceptual modeling framework could be adopted to discover dynamic land consequences and understand their responses for sustaining ecosystem services in the dominant agricultural region [16] and land transition information for sustainable management and development of land resources [17]. It has also been reported that the input data uncertainty has a higher influence on land use output and hence high-resolution geospatial inputs were recommended for crop and land management in this dominant agricultural region [18,19]. In most cases, the detrimental effect of temperature rise was observed even with elevated CO_2_ levels. Considerable spatial and temporal variations were also noted. Resilience development through climate change adaptation strategies is also a strong part of the challenges for crop production in Bangladesh [20]. Wheat was more susceptible to high temperature than rice and temperature increase generally reduces production across all scenarios [21]. Precipitation changes can have either a positive or a negative impact, with a high degree of uncertainty across Global Circulation Models (GCMs) [22]. Carbon dioxide impacts on crop production are positive and depend on the emissions pathway. Precipitation uncertainties from different GCMs and emissions scenarios are reduced when integrated across the large Ganges-Brahmaputra-Meghna Basins’ hydrology [23]. Irrigation requirements are likely to increase with regional variations in agricultural production [24].

Agriculture in southern Bangladesh is severely affected by sea level rise even when cyclonic surges are not fully considered, with impacts increasing under the higher emissions scenario. The impact of climate change on cereals and food security has been studied by several authors; however, no effort in using farming system approaches integrating cropping systems with other income-generating activities has been made for Bangladesh until now. This study was undertaken as a component of the IGB (Indo-Gangetic Basin) regional assessments following AgMIP protocols and integrated assessment procedures. It is expected that this integrated assessment of climate change impacts on agricultural systems would generate reasonable estimates to help policymakers to develop national and regional plans for projected future agricultural systems.

## 2. Materials and Methods

### 2.1. Description of Study Area

**Figure 1:**
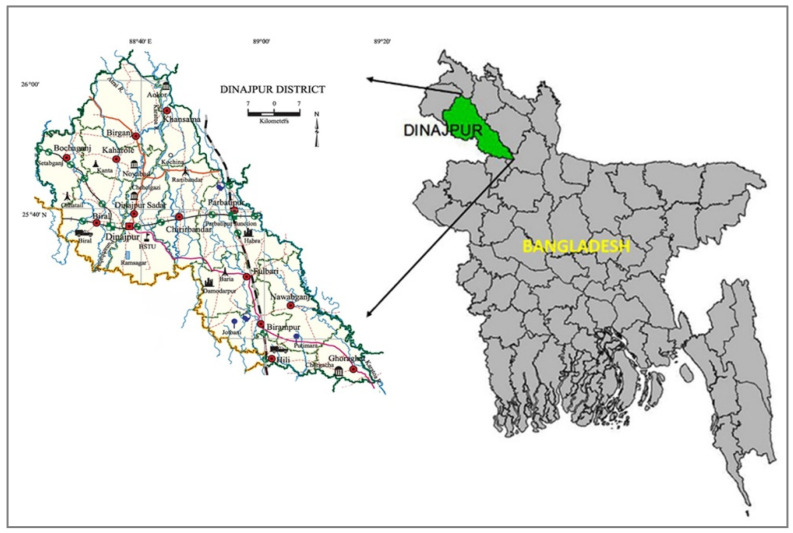
Location of the study.

The study area is located in the Dinajpur district (25°22’–26°06′ N, and 89°31′–88°38′ E), which is part of the Eastern Gangetic Plain region in Bangladesh (Figure 1). The total area of the Dinajpur district is 3438 km^2^ of which 78.87 km^2^ is forested [25]. Dinajpur is one of the most intensive agricultural areas among the crop production hubs in Bangladesh. The total cultivable cropped land was around 2707 km^2^ in 2014–2015 [26]. The general weather type of this region is humid and wet with subtropical climate variations identified as summer, monsoon, and winter seasons. The yearly mean highest temperature of the study location was around 33.5 °C and the lowest one was nearly 10.5 °C. The annual total rainfall was commonly 2540 mm. The dominant soil types of this region are divided into three categories, e.g., Piedmont plan, Tista Floodplain, and Barind Tract [27]. Based on flooding during the monsoon, the lands of Dinajpur can be divided into three distinct land types: High Land-HL (e.g., land higher than the normal flooding point), Medium High Land-MHL (e.g., land flooded up to 90 cm for a minimum of two weeks), and Medium Low Land-MLL (e.g., the land flooded between 90 to 180 cm for over two weeks) [27]. The texture of the topsoil (usually 0–15 cm) is primarily loam and silt loam, although it can also be sandy loam, silty clay loam, or clay loam [28].

### 2.2. Farming System Diagram

**Figure 2:**
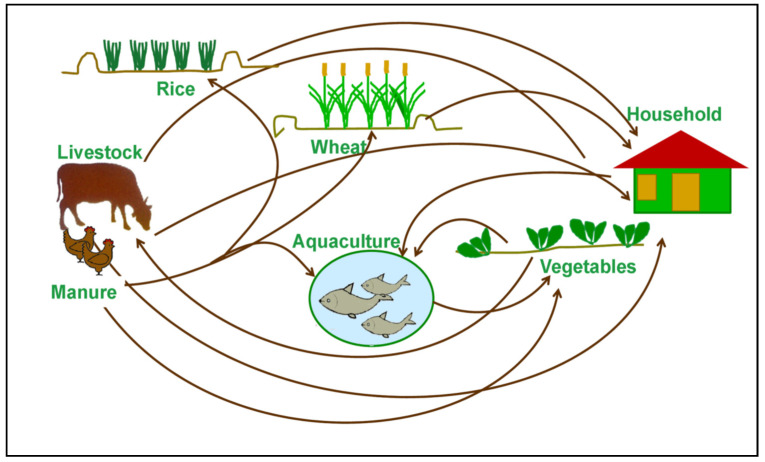
System diagram for study location.

Rice (*Oryza sativa*) is the most important commodity in terms of livelihood and food in the study area, followed by wheat (*Triticum aestivum* L.). In Bangladesh, rice is grown in three distinct seasons: Aus (Kharif-I), Aman (Kharif-II), and Boro (Rabi), while wheat is grown only during the winter (Rabi). The most dominant farming system of the study area was rice–wheat covering about 67% of the net sown area. The rice and wheat acreage in Dinajpur were 0.42 million hectare (Mha) and 0.019 Mha, respectively, with almost the same level of average productivity between rice and wheat (3.2 and 3.1 tha^−1^, respectively). Though livestock is an integral part of the farming system, this study focused on crops enterprise only. The cultivar used for rice was BR11 (T. Aman) cultivar which is grown during the wet season (Kharif-II). BR11 is the most popular variety for T. Aman season which is grown from 15th to 30th July. Usually, 25–35-day-old seedlings are transplanted with 20 cm row spacing. The crop’s lifecycle is 145 days. For wheat, BARI-Gom21 (Shatabdi) cultivar was used, which is grown during the winter season. The optimum sowing date for wheat is 15th November to 7th December with a lifecycle of 109–112 days. The yield potentials of BR11 and Shatabdi is 5.5 tha^−1^ and 3.5–5.0 tha^−1^, respectively [29]. For integrated assessment, a field survey was carried out during May–September 2019 to collect data from 50 randomly selected farms from four Upazilas (sub-districts) of the Dinajpur district to cover the major cropping pattern, e.g., the rice (Transplanted Aman)–wheat cropping pattern. All data were collected through a questionnaire survey directly from fifty sampled farms. The collected data were then cleaned, edited, standardized, and organized as per the prescribed format in spreadsheets to fulfil the requirements for running the crop and socio-economic models (Section 2.5). The farming system diagram of the study location is presented in Figure 2.

### 2.3. Representative Agricultural Pathways (RAPs) and Adaptation Package

The Representative Agricultural Pathways (RAPs) is an overall narrative description of a plausible future development pathway, and also contains key variables with qualitative storylines and quantitative trends, consistent with higher-level pathways (e.g., SSPs, global RAPs developed by the AgMIP Global Modeling Group). RAPs are translated into one or more scenarios (parameterizations) for The Tradeoff Analysis Model for Multi-dimensional Impact Assessment (TOA-MD) and crop models. These scenarios represent a set of technology and management adaptations to climate change. These scenarios, developed for specific RAPs, typically include changes in the types of crops or livestock produced and the way they are managed (e.g., use of fertilizers and improved crop cultivars). Procedures for RAP development are based on the stepwise process as described earlier [30].

The future trends in the agricultural system, and the RAP narratives were developed by the project team and were shared with the stakeholders. Several interactions were made with different stakeholders that included academicians from agricultural universities and scientists from the national agricultural research system to prepare the RAPs. Moreover, during the farm survey, interactions were held with 50 farmers from four sub-districts of the Dinajpur district, and their opinions about current climatic variability and how they perceive climate change and their anticipated impact on agriculture were recorded.

A combination of population growth, government subsidy on fertilizers, and improved economic performance is expected to cause a shift from agriculture to the service industry. From the historic data, it is observed that farm and family size is decreasing over time. The stakeholders agreed that farm size would decrease by 20 percent and family size would decrease by 10 percent during the next 30-year period [31]. The RAPS parameters and adaptation strategy used in the TOA-MD analysis are given below (Table 1).

To minimize the adverse impact of climate change on wheat production, several adaptation options were proposed by the participants in the stakeholders’ workshop. However, the following adaptation strategies were finalized: Modification of irrigation dates and amount of water for wheat. Advancement of sowing date by one week to avoid terminal heat stress for wheat. Improved fertilizer management and soil test-based application in both rice and wheat. Use of short-duration rice varieties so that advanced wheat planting can be accommodated. Out of the suggested adaptation measures, increasing the irrigation amount by 50 mm per irrigation for wheat was tested in this integrated assessment.

### 2.4. Data Collection and Methods of Study

#### 2.4.1. Soil Data Preparation

The soil profile-wise data were gathered from Reconnaissance Soil Survey Reports (Soil Resource Development Institute, Bangladesh). A total number of 45 soil series profiles were created as input for DSSAT models (e.g., AGMIP13001 to AGMIP13045). Examples from the Amnura soil profile are presented in Supplementary Tables S1 and S2. Four soil profiles, e.g., Amnura (AGMIP13028), Jamun (AGMIP13035), Ekdala (AGMIP13032), and Gangachara (AGMIP13033), were used for the simulation runs for Dinajpur.

#### 2.4.2. Observed Trends in Temperature and Precipitation

**Figure 3:**
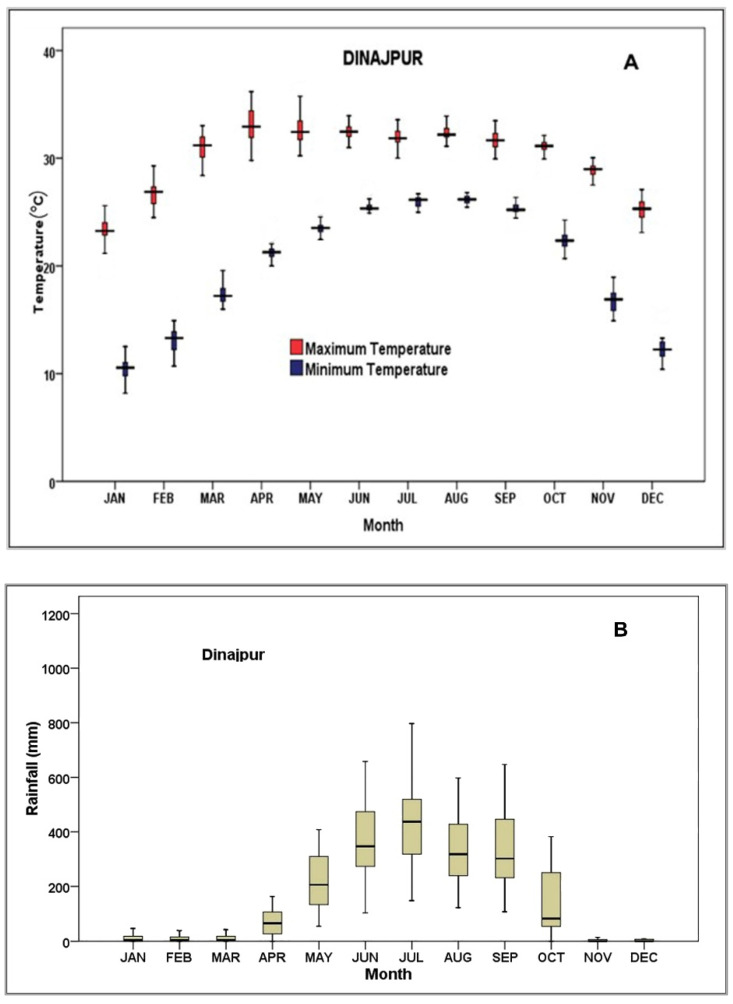
Monthly observed average (1990–2020) maximum and minimum temperature (**A**) and rainfall (**B**) of Dinajpur.

Historical time series (1990–2020) daily agro-meteorological data (daily maximum and minimum temperature, daily precipitation, and daily sunshine hour) were collected from the Bangladesh Meteorological Department (BMD, Supplementary Table S3). The sunshine hour data were converted to solar radiation (MJ m^−2^ d^−1^) as per the input format of the crop models as described by Allen et al. [32]. The input weather dataset was structured as per the model requirement. These were used as a historical climate dataset. The trends in temperature and precipitation observed data are presented in Figure 3.

#### 2.4.3. Climate Projections

**Figure 4:**
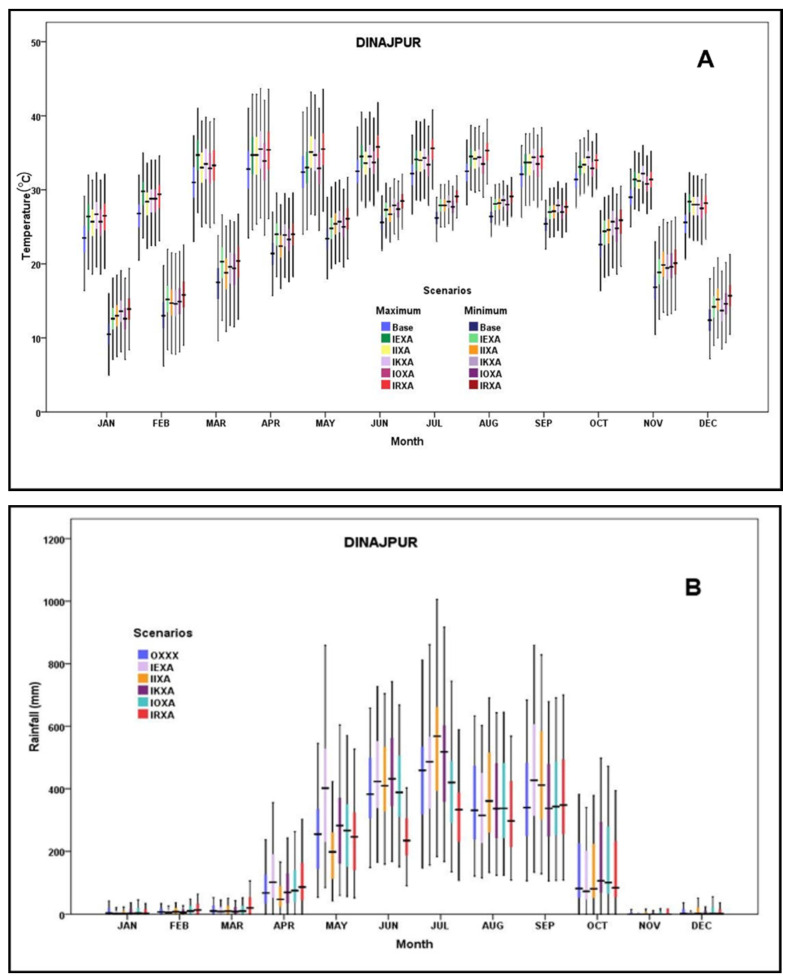
Predicted maximum and minimum temperatures (**A**) and rainfall (**B**) in Dinajpur.

Using the AgMIP Climate Scenario Generation Tools with R, total 20 Global Circulation Model (GCM) data were generated for RCP 8.5 (2040–2069, Mid-Century) based on the observed (OXXX) climate (30 years weather data (1990–2020-baseline)) data. Based on GCM climate change scenarios, five more scenarios, namely IEXA, IIXA, IKXA, IOXA, IRXA, for the median future scenarios were also generated for model runs. Here, the first letter I stands for RCP 8.5 (2040–2069, Mid-Century); E = CCSM4; I = GFDL-ESM2M; K = HadGEM2-ES; O = MIROC5; R = MPI-ESM-MR; type of scenario- X = Observations (no scenario); A = the mean change from GCM. In the case of RCP8.5 Mid-Century 2040–2069 scenarios, CO_2_ concentration was considered at 571 ppm [33]. The winter period (December, January, and February) showed a similar pattern regardless of GCMs. In this study, all the GCMs forecast higher summer temperature. Moreover, a reduced amount of rainfall was predicted during the winter season and higher during the monsoon period (mid-June to mid-October). Predicted maximum and minimum temperatures and rainfall in Dinajpur are presented in Figure 4.

**Figure 5:**
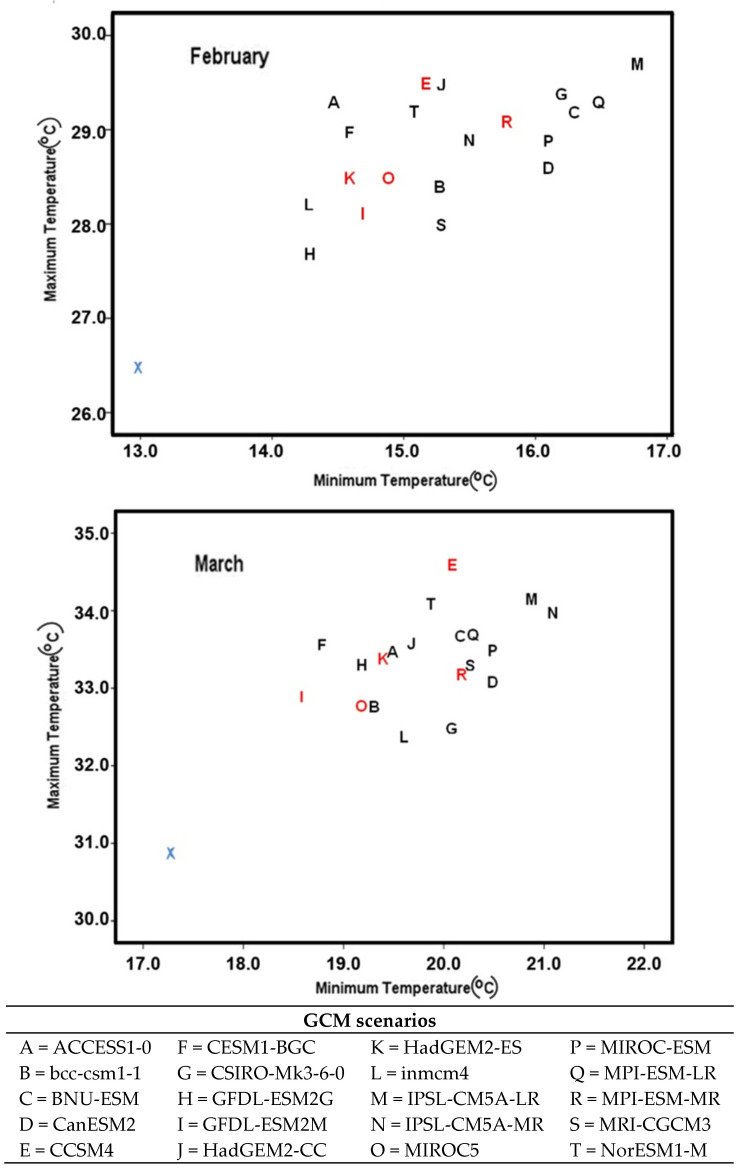
Maximum and minimum temperatures from 20 Mid-Century (RCP8.5) GCMs for Dinajpur (the letters in red are selected for the simulation runs, blue is the baseline, and the rest are different GCMs for RCP8.5).

From Figure 5, it is clear that all 20 Mid-Century scenarios projected higher maximum and minimum temperatures in February and March compared to the baseline time period (blue X). Again, March is warmer than February. The highest February maximum temperature (29.69 °C) is predicted by IPSL-CM5A-LR (M), and the highest March maximum temperature (34.58 °C) is predicted by CCSM4 (E); the rest of the GCMs are predicted above 30 °C.

### 2.5. Socio-Economic Analysis

Socio-economic data of 50 representative farm households were collected from Dinajpur in 2019. The average household size was 5.74 persons per house against the national average of 5.31 persons per house [34]. The average farm size was 0.90 ha. The average annual non-agricultural income was Bangladeshi taka (BDT) 59,600 farm^−1^, and the contribution of crop component was BDT 40,690 farm^−1^ year^−1^. The average plot size of rice was 0.65 ha. The majority of the farmers cultivated rice in medium high land (58%) followed by high land (26%) and medium low land (16%). Farmers applied 150.15 kg nitrogen, 69.14 kg TSP, 51.37 kg MoP (Muriate of potash) per hectare, which is higher than the recommended doses of fertilizers for T. Aman rice (90–120 kg N, 80–100 kg TSP and 80–120 kg MoP, and 50–72 kgha^−1^ gypsum (FRG, 2012)). The crop was grown in rainfed conditions. The farmers obtained 3826 kgha^−1^ grain yield which was a little higher than the five-years mean yield of rice (3801 kgha^−1^) in Dinajpur. The average plot size of wheat was 0.40 hectares. Most of the farmers cultivated wheat in medium high lands (58%) followed by high land (28%) and medium low land (16%). The input use per hectare by the farmers was 2139 kg farmyard manure (FYM), 86.5 kg N, 84.3 kg TSP, 78.2 kg MoP, 20.2 kg gypsum, 1.83 kg zinc sulphate, and 9.09 kg borax. The recommended fertilizer doses for wheat were 81–99 kg N, 140–180 kg TSP, 40–45 kg MoP, and 110–120 kg gypsum per hectare (BARI, 2019). The farmers obtained 3346 kgha^−1^ grain yield, which was much higher than the five-year mean yield of wheat (2497 kgha^−1^) in Dinajpur.

### 2.6. Crop-Model Calibration (APSIM and DSSAT)

In this study, two crops (rice and wheat) were modeled. The crop models used are CERES-Rice and CERES-Wheat for DSSAT Ver. 4.5.1.023 –Stub [35] and APSIM 7.5-Oryza and APSIM-Wheat [36]. The Agricultural Production Systems Simulator (APSIM) is a software package that facilitates sub-modules combined to simulate agricultural systems [13]. The APSIM simulator has several modules categorized as Plant-Soil-Atmosphere and crop management operations. The model can simulate the crop growth stage developments, soil processes, and crop management options. The APSIM model requires the input dataset of weather, soil, and crop management development on a daily basis to run the simulation outputs. The DSSAT cropping model is a computer-based crop growth application that can simulate growth, development, and yield of crop growing under defined managements over time [14,15]. The DSSAT model requires databases of daily basis weather data, soil, crop management conditions, and cultivar specific genotypes to simulate and compare results with observed estimates. Estimated genetic coefficients for the selected cultivars are presented in Table 2.

The calibration outputs for rice (APSIM and DSSAT) and wheat (APSIM and DSSAT) are presented in Table 3 and Table 4, respectively. Supplementary Figures S1 and S2 depict the calibration and validation outputs (observed and simulated) using the APSIM model for the rice (BR11-T. Aman) and wheat (Shatabdi) cultivars, respectively.

### 2.7. Validation of Crop Simulation Models

The crop model calibration is the process of comparison between the observed and simulated values that confirmed the acceptable estimates of the model outputs. Crop models require cultivar specific genetic coefficients to simulate performance of diverse genotypes under different soil, weather and management conditions (crop growth, development, and grain production). The calibration outputs and the validation of different crop simulation models need to operate in different ways. Two statistical criteria were used to validate the model: the normalized root mean square error (NRMSE) and the root mean square error (RMSE) [37].

(1) $$\mathrm{RMSE}=\sqrt{\frac{\sum_{i=1}^{n}{\left(S_{i}-O_{i}\right)}^{2}}{n}}$$

(2) $$\mathrm{NRMSE}=100\frac{\sqrt{\frac{1}{n}\sum_{i=1}^{n}{\left(S_{i}-O_{i}\right)}^{2}}}{\mathrm{Omax}-\mathrm{Omin}}$$

where *s* are the simulated values, *o* are the observed values, and *n* are the number of data points. Omax and Omin are the maximum and minimum values of the observations, respectively.

## 3. Results

### 3.1. Evaluation of Crop Models (APSIM and DSSAT)

**Figure 6:**
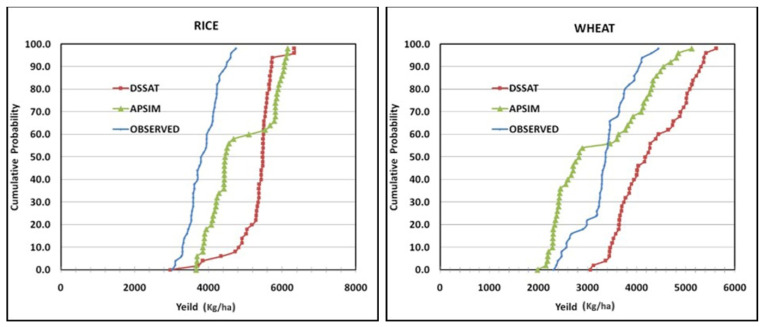
Cumulative distribution functions for observed rice and wheat yields, and APSIM and DSSAT simulated rice and wheat yields.

Intercomparison was made between two rice models (APSIM-Rice and DSSAT-CERES-Rice). Compared to the observed yields, higher yields were predicted by both models for the Dinajpur location. The ambiguity in yields related to different farms was greater with APSIM model predictions compared to the DSSAT model. However, both the models overvalued the simulated crop yields. Higher crop yields were predicted by the DSSAT-Wheat model in comparison with the APSIM-Wheat model. The APSIM-wheat model predicted less than 3500 kgha^−1^ at 55% cumulative probability, which was lower than the observed yield. The uncertainty of cumulative probability was higher with APSIM in simulated crop yields among different farms compared to DSSAT (Figure 6). Higher simulated yields compared to farm survey yields are expected since neither models considered pest and disease effects.

To evaluate the impact of future climate scenarios with APSIM and DSSAT rice and wheat models, the yield performance of the 30-year baseline weather dataset was used to simulate 30 years of future climate scenarios. There was a total of six sets of climate data, namely (OXXX) for the current climate, and IEXA, IIXA, IKXA, IOXA, and IRXA future scenarios were predicted for CO_2_ at 571 ppm level.

The input files for the simulation were generated based on the following parameters.

Rice (rainfed): The simulation runs were made for Dinajpur location using DSSAT CERES-Rice and APSIM-Rice models. The rice cultivar was BR11 (T. Aman), which was sown from 31 May to 10 August of 2018. The cultivation depth was 5 cm. Final crop density was 25 plants m^−2^ and three plantshill^−1^. Nitrogen was applied as basal at sowing @27–40.5 kgha^−1^ with a placement depth of 10 cm. Most of the farmers applied nitrogen in two splits @39–46 kgha^−1^ and 39–46 kgha^−1^ as top-dressing at 15 and 45 days after sowing, respectively.

Wheat (irrigated): The simulations were carried out for the Dinajpur location with DSSAT CERES-Wheat and APSIM-Wheat models. The wheat cultivar was BARI Gom21 (Shatabdi). The sowing period of the wheat cultivar was from 11 November to 07 December of 2018 with a cultivation depth of 5 cm and crop density of 240 plantsm^−2^. Nitrogen was applied as basal at sowing @30–40 kgha^−1^ with a placement depth of 10 cm. Most of the farmers applied nitrogen in two splits @25–35 kgha^−1^ and 25–40 kgha^−1^ as top-dress at 25 and 55 days after sowing, respectively; and three irrigations (flood method) at 20, 45 and 75 days after sowing to maintain the soil moisture at field capacity level.

The simulated yields are presented with box and whisker plots. The box represents data between the 25th and 75th percentiles. The center black horizontal line across the box is the median at 50% of yields, and the whiskers (error bars) above and below the box indicate the 95th and 5th percentiles.

**Figure 7:**
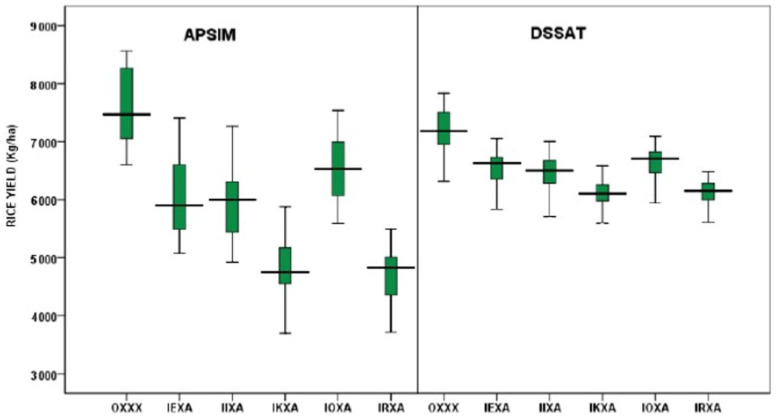
Simulated rice grain yields (APSIM and DSSAT) compared with baseline and future scenarios.

For the single treatment, the DSSAT CERES-Rice and APSIM-Rice models were simulated with 30 seasons of baseline weather (base) and five different climate scenarios (IEXA, IIXA, IKXA, IOXA, and IRXA). APSIM and DSSAT rice models predicted lower yields for all the GCM scenarios compared to the baseline, but the magnitude of yield decreases was small in the case of DSSAT, and a higher yield variability was noted in the case of APSIM (Figure 7). Only higher yields were predicted by the IOXA scenario. The lowest yields were predicted by the IKXA scenario for both the models (Figure 7). On the other hand, APSIM predicted lower yields for all the GCM scenarios compared to the baseline, but the magnitude of yield decreases was quite high. The variability observed in the yields in different farms may be due to variable fertilizer and water management caused by variable availability of rainwater and temperature increase.

The maximum rice yield values predicted by APSIM for OXXX (Historical), IEXA, IIXA, IKXA, IOXA, and IRXA scenarios were 8557, 7408, 7267, 5880, 7538, and 5492 kgha^−1^, while the minimum values were 6607, 5071, 4920, 3697, 5586, and 3718 kgha^−1^ with average values of 5904, 7472, 6003, 4749, 6533, and 4829 kgha^−1^, respectively (Figure 7). In the case of DSSAT, the predicted maximum values were 7832, 7053, 7005, 6584, 7095, and 6483 kgha^−1^ for OXXX (Historical), IEXA, IIXA, IKXA, IOXA, and IRXA scenarios, respectively. However, the minimum yields were 3756, 3703, 3640, 3652, 3744, and 4112 kgha^−1^ with average yield values of 7185, 6630, 6503, 6107, 6710, and 6152 kgha^−1^ (Figure 7).

**Figure 8:**
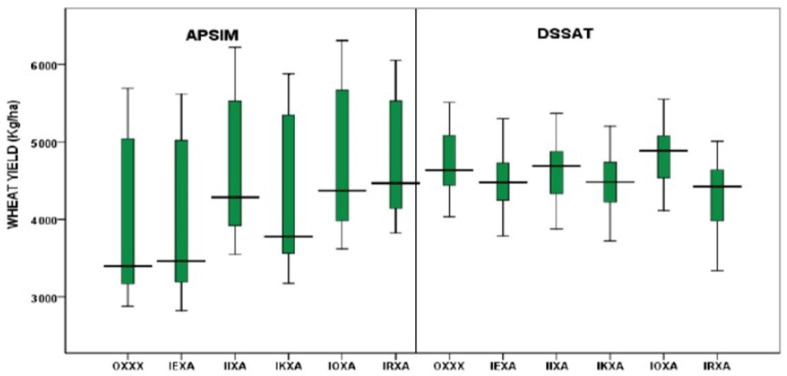
Simulated wheat yields (APSIM and DSSAT) compared with baseline and future scenarios.

Similarly, the APSIM-Wheat and the DSSAT CERES-Wheat models were simulated with 30 seasons of baseline weather (base) and five different climate scenarios (IEXA, IIXA, IKXA, IOXA, IRXA). For both DSSAT and APSIM models, predicted yields were higher for all climate scenarios (Figure 8). In the case of the DSSAT model, future climate scenarios IEXA, IKXA, and IRXA have more adverse climatic effects on wheat yield compared to the baseline scenario. In contrast, the future climate scenarios showed positive effects on wheat yield for the APSIM model (Figure 8).

The APSIM model projected maximum wheat yield for OXXX, IEXA, IIXA, IKXA, IOXA, and IRXA scenarios were 5692, 5624, 6223, 5879, 6308, and 6054 kgha^−1^, and the minimum were 2878, 2825, 3547, 3175, 3621, and 3824 kgha^−1^ with average yields of 3464, 3398, 4286, 3780, 4369, and 4471 kgha^−1^, separately (Figure 8).

At the same time, the DSSAT model anticipated that the maximum wheat yields for OXXX, IEXA, IIXA, IKXA, IOXA, and IRXA were 5507, 5299, 5369, 5204, 5548, and a minimum of 5009 kgha^−1^, and 4036, 3470, 3452, 3380, 3649, and 3338 kgha^−1^ with average projected yields were 4637, 4481, 4691, 4485, 4885, and 4426 kgha^−1^ (Figure 8).

To offset the impact of climate change and reduce the variability in wheat yields, an adaptation strategy was incorporated in this study. Although there were several adaptation strategies suggested in the RAP development workshop, only one, e.g., increasing the irrigation amount by 50 mm per irrigation for wheat, was used. Accordingly, both APSIM and DSSAT wheat models were used to generate outputs under five different CGMs.

**Figure 9:**
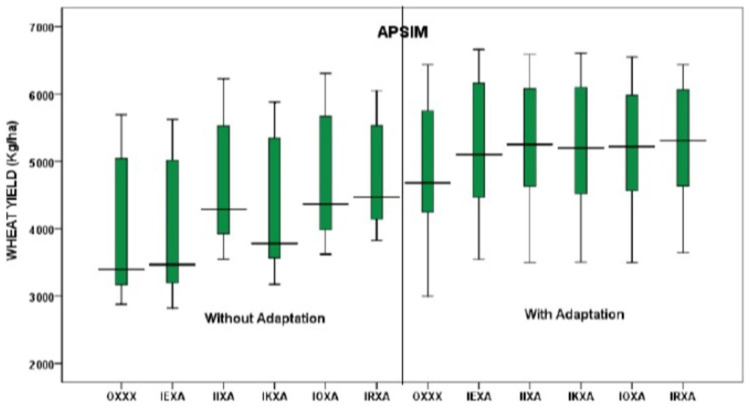
Simulated wheat yields (APSIM) without and with adaptation.

The APSIM model showed reduced variability in yields with adaptation strategy irrespective of GCMs. In the case of the baseline (OXXX) scenario, the yields were 2878 to 5692 kgha^−1^ without adaptation and 2998 to 6434 kgha^−1^ with adaptation. Among all scenarios, the IRXA scenario showed the maximum average yield in both without and with the adaptation strategy (Figure 9).

**Figure 10:**
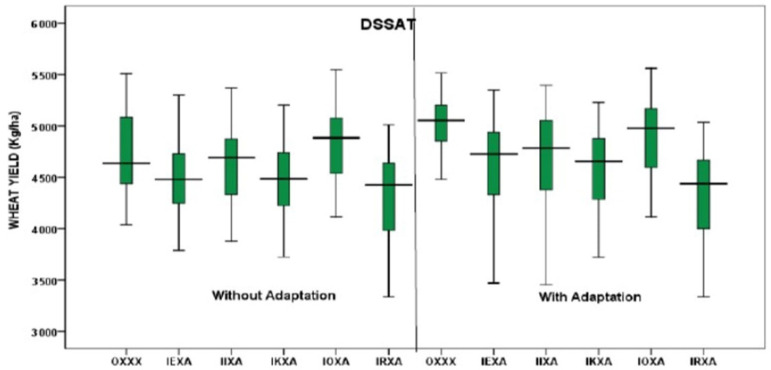
Simulated wheat yields (DSSAT) without and with adaptation.

Correspondingly, the DSSAT model indicated that projected median yields were raised with the adaptation in all GCMs and reduced variability in yields. In the case of DSSAT, the predicted minimum yields for the historic baseline (OXXX) scenario without adaptation was 4036 kgha^−1^ and with adaptation was 4112 kgha^−1^ (Figure 9). The maximum yield without adaptation was 5507 kgha^−1^ and with adaptation was 5514 kgha^−1^, respectively. The IRXA scenario showed the lowest median yields without and with adaptation of DSAAT compared to APSIM. The simulated minimum and maximum yield predictions for the IRXA scenario differed between 3338 and 5009 kgha^−1^ without adaptation, and between 3338 and 5034 kgha^−1^ with adaptation (Figure 10).

### 3.2. Impact of Climate Change on Crop Production

The sensitivity of the current production system under five different climatic scenarios is presented in Table 5. The current production system, under all five climate change scenarios, shows that the mean yield of rice would decline by 13.6–38.2 percent (with APSIM) and 7.4–14.7 percent (with DSSAT), respectively (Table 5). On the other hand, mean wheat yield is likely to increase (0.8–21.8%) under all five climate scenarios (with APSIM). However, DSSAT estimates show mixed results—a decline in wheat yield (2.9–8.8%) under four climate scenarios (IEXA, IIXA, IKXA and IRXA), and a marginal increase (1%) under climate scenario IOXA.

The current production system is vulnerable to climate change as it shows decline (4.6–17.8%) in mean net farm returns (APSIM model) under all but one climate scenario (IOXA) which shows marginal increase (0.74%) in net farm returns. The extent of losses in net farm returns varies from 4–11.6% (DSSAT model). Consequently, the per capita income under climate change declines (0.8–3.9%) in all the scenarios except one scenario (IOXA for APSIM model) which shows a marginal increase (0.16%). Though changes in the poverty levels are minimal, there is a large proportion of the population (48–80%) which is adversely affected by climate change (Table 5).

### 3.3. Impact of Climate Change on Future Agricultural Systems

**Figure 11:**
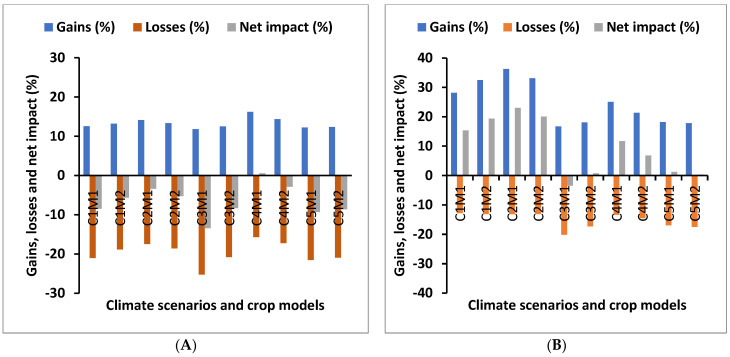
Comparison between gains, losses, and net impacts under current agricultural production system and future agricultural production system by different climate scenarios and crop models (C1–C5 = Climate models; C1 = CCSM4, C2 = GFDL–ESM2M, C3 = HadGEM2–ES, C4 = MIROC5, C5 = MPI–ESM–MR; M1 = crop model (APSIM), M2 = crop model (DSSAT)). (**A**) Gains, losses, and net impact under current climate. (**B**) Gains, losses, and net impact under future climate.

Using RAP parameters and country-specific estimates of productivity and price trends for rice and wheat from global models, TOA-MD analysis was done to study the impact of climate change on future agricultural production systems. In the case of rice, larger yield decline (14–38%) was predicted by the APSIM model than that by the DSSAT model (7–15%). Interestingly, wheat yield was likely to increase (0.8–22%) under all climate scenarios (APSIM model); however, DSSAT estimates show decline in wheat yields (3–9%), except under one climate scenario (IOXA) which shows a marginal increase (1%) in wheat yields (Table 6). Interestingly, the mean returns per farm were projected to increase under all but one climate scenario (IKXA, APSIM model) in future in the range of 2–29% and 0.5–26% for APSIM and DSSAT, respectively (Table 6). It appears that the decline in rice yields is more compensated by the increase in wheat yields (APSIM) and higher growth trend in yield and prices of rice and wheat along with a doubling of off-farm income in future agricultural production systems. The cumulative effect is strong enough to offset the decline in productivity (rice) and acreage under the rice–wheat system. Though increase in per capita income is quite low (0.07–4.8%), except IKXA- APSIM model (−0.78%), it substantially reduces the proportion of the population which is adversely affected by climate change. Total gains, losses, and net impact on farm population are shown in Figure 11A,B. Though it appears a small change in per capita income, it brings about 37–39% of the population out of poverty (Table 6).

### 3.4. Benefits of Climate Change Adaptations

Though parameters derived from the RAPs scenario and the growth trend in price and productivity of the rice–wheat system resulted in overall increase in net farm returns, yet more than half of the population remains below the poverty line. So, it would be imperative to assess the impact of adaptation measures on net farm income and livelihoods. In this study, the adaptation strategy was tested for wheat crop only, wherein irrigation amount was increased by 50 mm per irrigation. The adaptation package for wheat results in higher increased (10–32%) in wheat yields with APSIM model in comparison to DSSAT model (1–3% only) under five climate scenarios (Table 7). Consequently, the increase in mean net farm returns was larger (5–19%) with APSIM than that for DSSAT (7–10%). Overall, the per capita income increased up to 3.6% only, and the poverty rate declined marginally (Table 7). It is obvious because wheat acreage is less than that of rice, which is the most important crop and affects the livelihoods of most of the population.

## 4. Discussion

Crop simulation model evaluation is a fundamental activity to compare the observed and simulated growth and development of crops performed by different models [24]. APSIM and DSSAT crop models are widely used for agronomic crop simulation performance for development and yields predictions [30]. Based on the input dataset and the climatic stresses and uncertainty, both the APSIM and DSSAT model can perform different simulation results for rice and wheat from the same location [32]. In the case of wheat, APSIM showed higher sensitivity and lower yield prediction than the observed farm yields and DSSAT estimated higher yield in our study which also studied in different parts of China [33]. Moreover, the ambiguity in yield prediction with different sites was more in the case of APSIM compared to DSSAT in Dinajpur. The variations in yield predictions between the APSIM and DSSAT models could be due to the exclusion of pest and disease infestation and damage [31]. In the current study, the sensitivity of both the APSIM and DSSAT crop models estimated the lower yield predictions by 13.6–38.2% (APSIM) and 7.4–14.7% (DSSAT) for rice under different climate scenarios (Table 7). The current crop production system in most parts of the Indo-Gangetic belt showed a decreasing rice yield trend for both APSIM and DSSAT models [36]. Moreover, increasing CO2 concentration (571 ppm) will influence the total global average temperature, which will affect rice and wheat yields [34]. Climate change has a significant impact on agricultural land use diversity, cropping area, and production based on the shifting pattern of rainfall and its fluctuation, temperature, flooding risk, agroecological features of different regions, humidity, and sunshine hours [38,39]. In response to climate change, farmers may utilize more inputs, which could have an impact on future agricultural productivity and production efficiency as well as on human health and the environment [40]. Other studies noted that due to the use of pesticides and chemical fertilizers, water extraction for irrigation, crop intensification, and CO_2_ emissions can enhance the uncertainty in sustainable agricultural production (rice–wheat cropping system) in Bangladesh [41]. This declined rice production will affect the net farm returns under climate change scenarios [35]. In contrast, mean wheat yield is likely to increase by 0.8–21.8 per cent (APSIM) and mixed results–decline in wheat yield by 2.9–8.8 per cent (DSSAT). A recent study also suggested that both APSIM and DSSAT yield variabilities for wheat can be managed by strategic management of fertilizer applications in some cases [36]. Socio-economic analysis for the current study estimated the impact of climate change on future agricultural production systems on farm level. In the case of rice, yield decline was predicted by APSIM and DSSAT model in all future climate scenarios. Remarkably, wheat yield is estimated to rise under all climate scenarios by the APSIM model, while DSSAT predicted decline in wheat yields. The cumulative effect is enough to balance the decline in productivity (rice) and land use under the rice–wheat system. The impact of future climate change events on the rice–wheat cropping system has mixed magnitude on rural farm level production [42]. Using crop models can help farmers make decisions about how to manage their crops to reduce climatic risk and make the most use of limited resources [43]. Crop yield predictions, using future climate scenarios and various crop models from the same site where all other necessary parameters for cropping are known, can bring the chance to compare the selection of crop model for predictable crop yield based on the impacts of climate change [14,44,45,46]. In this study, the adaptation strategy was analyzed for wheat crop with irrigation amount increased by 50 mm per irrigation which resulted 10–32 percent higher yield with APSIM compared to the same amount applied to project with DSSAT. The planting time, irrigation schedule, and amount of irrigation along with climate change also influenced the growth and development of the crops. Improved fertilizer method, sowing density, and climate resilient cultivar can also be considered as major adaptation strategies for rice and wheat cropping systems [24,47]. To combat the diverse impact of climate change, maintaining the irrigation water use efficiency and introducing drought resistant cultivars plays a significant role [48]. The current study revealed that the benefits of the climate change adaptation strategy can increase the per capita income by up to 3.6% on the farm level and thus could potentially reduce the poverty rate. It is to be noted that adaptation is crucial and responses to climate change need to be well-integrated with poverty reduction strategies to achieve sustainable development. Moreover, climate change adaptation strategies exert direct and immediate impacts on the poor by affecting factors that condition poverty reduction, such as economic growth.

## 5. Conclusions

Since rice is the most important crop for the population, there is a need to test the adaptation strategy for rice for a more realistic integrated assessment. This study confirmed the development and potential application of an integrated approach of climate change impact assessment. From the climatic perspective, the findings of the study suggested that the rice–wheat cropping system can be adjusted by some adaptation strategies. The duration of rice cropping can be minimized by using the short-term high yielding rice varieties to adjust the wheat sowing timing 7 days earlier than the traditional mid-November to 1st week of November. Shifting the wheat sowing date will minimize the monsoon threat on the wheat ripening stage, which will ensure the quality of grain and the storage life. Additionally, increasing the irrigation amount by 50 mm in wheat cultivation increased the yield. However, this study could not include other enterprises (livestock, fishery, etc.) in the assessment. Therefore, a holistic assessment of climate change on future production systems would only be possible by including all the relevant enterprises of the agriculture sector and testing the most effective adaptation strategy. Lastly, a methodological framework should be developed for upscaling the integrated assessment for the entire Indo-Gangetic Basin.

## Figures and Tables

**Table 1:** RAPs parameters for Bangladesh used in the model and adaptation strategy for wheat.

Parameters	Direction	Magnitude (%)
Farm size	Decrease	20
Family size	Decrease	10
Variable costs of production	Increase	140
Off farm income	Increase	100
Rice yield trend without CC	Increase	112.5
Rice price trend without CC	Increase	127.0
Rice price trend with CC	Increase	150.3
Wheat yield trend without CC	Increase	111.2
Wheat price trend without CC	Increase	126.8
Wheat price trend with CC	Increase	156.8
Adaptation strategy for wheat		
Irrigation in wheat	Increase	50 mm per farm

**Table 2:** Experimental dataset for genetic coefficient calculation for rice and wheat.

Rice	Wheat
Sowing date: 31 May 2018	Sowing date: 24 November 2018
Transplanting: 5 July 2018	Sowing density (plants m^−2^): 240
Sowing depth (cm): 5 cm	Sowing depth (cm): 5
Row spacing (cm): 20 cm	Row spacing (cm): 20
Fertilizer and irrigation management:	Fertilizer and irrigation management:
N fertilizer: urea N	N fertilizer: urea N
Applied N (kgha^−1^): 90	Applied N (kgha^−1^): 120 (2 split)
1st N application amount (kgha^−1^): 30	1st N application amount (kgha^−1^): 80 (as basal)
2nd N application amount (kgha^−1^): 30	2nd N application amount (kgha^−1^): 40 (14 December 2018)
3rd N application amount (kgha^−1^): 30	1st irrigation: 14. December 2018
	2nd irrigation: 31 December 2018
	3rd irrigation: 24 January 2019
	4th irrigation: 7 February 2019

**Table 3:** Calibration outputs (Rice-APSIM and DSSAT) for cultivar BR11.

Stage Code		Date (Comparison)		
	Stage	Observed	Simulated (APSIM)	Simulated (DSSAT)
1	Sowing	31 May 2018	31 May 2018	2 June 2018
2	Transplanting	5 July 2018	5 July 2018	15 July 2018
3	End of juvenile	10 August 2018	10 August 2018	21 August 2018
4	Floral initiation	7 October 2018	3 October 2018	20 September 2018
5	Flowering	25 October 2018	25 October 2018	25 October 2018
6	Maturity	24 November 2018	24 November 2018	23 November 2018
7	Grain yield (kg ha^−1^)	4610	4743	6811
8	Biomass yield (kg ha^−1^)	10,310	13,212	14,171

**Table 4:** Calibration outputs (Wheat-APSIM and DSSAT) for cultivar Shatabdi.

Stage Code		Date (Comparison)		
	Stage	Observed	Simulated (APSIM)	Simulated (DSSAT)
1	Sowing	24 November 2018	24 November 2018	24 November 2018
2	Germination	25 November 2018	25 November 2018	25 November 2018
3	Emergence	3 December 2018	29 November 2018	27 November 2018
4	End of juvenile	15 December 2018	15 December 2018	16 December 2018
5	Floral initiation	17 January 2019	17 January 2019	20 January 2019
6	Flowering	31 January 2019	31 January 2019	31 January 2019
7	Start grain fill	6 February 2019	6 February 2019	9 February 22019
8	End of grain fill	13 March 2019	11 March 2019	11 March 2019
9	Maturity	14 March 2019	13 March 2019	13 March 2019
	Grain yield (kg ha^−1^)	5200	4820	4809
	Biomass yield (kg ha^−1^)	10,000	8203	10,378

**Table 5:** Climate sensitivity for rice–wheat system in Dinajpur, Bangladesh.

Parameters	IKXA		IOXA		IRXA		IEXA		IIXA	
	APSIM	DSSAT	APSIM	DSSAT	APSIM	DSSAT	APSIM	DSSAT	APSIM	DSSAT
Observed average crop yield (Rice) (kg ha^−1^)	3681	3725	3598	3637	3524	3589	3614	3681	3553	3634
Average crop yield change (Rice) (%) [defined as: (average relative crop yield − 1) × 100]	−37.40	−14.70	−13.60	−7.40	−38.17	−13.40	−20.30	−8.80	−21.70	−10.10
Observed average crop yield (Wheat) (kg ha^−1^)	2789	2827	2852	2889	2798	2841	2837	2894	2817	2910
Average crop yield change (Wheat) (%) [defined as: (average relative crop yield − 1) × 100]	9.00	−6.00	20.00	1.00	21.80	−8.80	0.80	−6.00	18.70	−2.90
Losers (%)	80.10	71.34	48.51	58.00	73.39	72.03	71.54	65.31	59.31	64.25
Gains (% average net farm returns)	11.78	12.48	16.23	14.33	12.24	12.35	12.56	13.19	14.10	13.35
Losses (% average net farm returns)	−25.23	−20.79	−15.69	−17.22	−21.54	−20.96	−21.04	−18.87	−17.47	−18.60
Observed net earnings without climate change (BDT farm^−1^)	16,903	16,903	16,903	16,903	16,903	16,903	16,903	16,903	16,903	16,903
Observed net earnings with climate change (BDT farm^−1^)	13,887	15,001	17,029	16,233	14,783	14,936	14,964	15,595	16,122	15,690
Observed per-capita earnings without climate change (BDT)	14,795	14,795	14,795	14,795	14,795	14,795	14,795	14,795	14,795	14,795
Observed per-capita earnings with climate change (BDT)	14,211	14,427	14,819	14,665	14,384	14,414	14,419	14,541	14,643	14,560
Observed poverty rate without climate change (%)	96.07	96.07	96.07	96.07	96.07	96.07	96.07	96.07	96.07	96.07
Observed poverty rate with climate change (%)	96.58	96.40	96.05	96.19	96.43	96.41	96.41	96.30	96.21	96.28

Note: Exchange rate: USD 1 = BDT 84.86. Poverty line = USD 1.25.

**Table 6:** Impact of climate change in rice–wheat system in Dinajpur, Bangladesh.

Parameters	IKXA		IOXA		IRXA		IEXA		IIXA	
	APSIM	DSSAT	APSIM	DSSAT	APSIM	DSSAT	APSIM	DSSAT	APSIM	DSSAT
Projected average yield (Rice) (kgha^−1^)	4537	4583	4561	4625	4681	4711	4516	4584	4592	4648
Average yield change (Rice) (%) [defined as: (average relative yield − 1) × 100]	−37.40	−14.70	−13.60	−7.40	−38.17	−13.40	−20.30	−8.80	−21.70	−10.10
Projected average yield (Wheat) (kgha^−1^)	3248	3356	3254	3315	3314	3357	3311	3406	3451	3487
Average yield change (Wheat) (%) [defined as: (average relative yield − 1) × 100]	9.00	−6.00	20.00	1.00	21.80	−8.80	0.80	−6.00	18.70	−2.90
Losers (%)	58.27	48.15	24.33	33.59	46.84	49.26	19.03	15.60	12.89	15.03
Gains (% average net farm returns)	16.68	18.05	25.03	21.31	18.20	17.82	28.13	32.50	36.23	33.11
Losses (%average net farm returns)	−20.17	−17.31	−13.34	−14.50	−16.94	−17.52	−12.78	−13.13	−13.19	−13.10
Projected net earnings without climate change (BDTfarm^−1^)	23,000	23,000	23,000	23,000	23,000	23,000	23,001	23,006	23,017	23,008
Projected net earnings with climate change (BDTfarm^−1^)	21,897	23,235	26,609	25,134	23,401	23,094	27,680	28,836	29,864	29,017
Projected per-capita earnings without climate change (BDT)	30,531	30,531	30,531	30,531	30,531	30,531	30,531	30,532	30,535	30,533
Projected per-capita earnings with climate change (BDT)	30,294	30,582	31,306	30,989	30,617	30,551	31,536	31,784	32,005	31,823
Projected poverty rate without climate change (%)	58.74	58.74	58.74	58.74	58.74	58.74	58.74	58.74	58.74	58.74
Projected poverty rate with climate change (%)	59.11	58.68	57.60	58.07	58.62	58.72	57.25	56.88	56.55	56.82

Note: Exchange rate: USD 1 = BDT 84.86. Poverty line = USD 1.25.

**Table 7:** Impacts of adaptation in rice–wheat system in Dinajpur, Bangladesh.

Parameters	IKXA		IOXA		IRXA		IEXA		IIXA	
	APSIM	DSSAT	APSIM	DSSAT	APSIM	DSSAT	APSIM	DSSAT	APSIM	DSSAT
Projected average yield without adaptation (Rice) (kg farm^−1^)	2261	3041	3093	3306	2225	3085	3605	4138	3557	4076
Average yield change (Rice) (%) [defined as: (average relative yield − 1) × 100]	0	0	0	0	0	0	0	0	0	0
Projected average yield without adaptation (Wheat) (kgfarm^−1^)	3558	2953	3200	2930	3195	2910	4284	3325	3633	3265
Average yield change (Wheat) (%) [defined as: (average relative yield − 1) × 100]	22.30	2.10	10.30	1.30	10.10	0.70	32.20	3.30	12.50	1.50
Adoption rate (%)	63.46	58.17	41.04	50.12	37.69	59.35	74.14	58.34	44.51	53.91
Projected net earnings without adaptation (BDTfarm^−1^)	21,897	23,235	26,609	25,134	23,401	23,094	27,682	28,836	29,864	29,017
Projected net earnings with adaptation (BDT farm^−1^)	24,723	25,611	28,144	27,108	24,606	25,562	32,986	31,846	31,823	31,620
Projected per-capita earnings without adaptation (BDT)	30,294	30,582	31,306	30,989	30,617	30,551	31,536	31,784	32,005	31,823
Projected per-capita earnings with adaptation (BDT)	30,901	31,092	31,635	31,413	30,876	31,081	32,675	32,430	32,425	32,382
Projected poverty rate without adaptation (%)	59.11	58.68	57.60	58.07	58.62	58.72	57.25	56.88	56.55	56.82
Projected poverty rate with adaptation (%)	58.21	57.92	57.11	57.44	58.25	57.94	55.58	55.93	55.94	56.00

Note: Exchange rate: USD 1 = BDT 84.86. Poverty line = USD 1.25.

## Data Availability

Not applicable.

## Supplementary Materials

The following supporting information can be downloaded at: https://www.mdpi.com/article/10.3390/ijerph192315829/s1.
