# Supplementary material for: Climate Change Impacts Assessment Using Crop Simulation Model Intercomparison Approach in Northern Indo-Gangetic Basin of Bangladesh
Source: Int J Environ Res Public Health. 2022 Nov 28;19(23):15829. doi: 10.3390/ijerph192315829 (PMC9738293; doi:10.3390/ijerph192315829)
Supplement: Supplementary file 1 [file ijerph-19-15829-s001.zip › ijerph-1984848-supplementary.pdf]

Supplementary File

Title: Climate change impacts assessment using crop simulation model intercomparison approach in northern Indo-Gangetic Basin of Bangladesh

Table S1. Soil properties of Amnura series input for DSSAT model

Layer Depth (cm)	LL, Lower limit (cm^3 cm^{-3})	DUL, Drained Upper limit (cm^3 cm^{-3})	SAT, Upper limit sat. (cm^3 cm^{-3})	RGF, Root Growth factor	BD, Bulk density (g cc^{-1})	pH in water	CEC (cmol kg^{-1})
10	0.118	0.289	0.511	1	1.21	4.6	5.7
15	0.105	0.261	0.491	1	1.27	5.4	5.3
27	0.141	0.293	0.482	0.657	1.3	6	11.2
48	0.127	0.283	0.493	0.472	1.27	5.6	12.1
76	0.152	0.31	0.485	0.289	1.29	5.6	11.4
99	0.188	0.352	0.488	0.174	1.28	5.6	12.3
132	0.178	0.341	0.496	0.099	1.26	5.8	11.1
165	0.152	0.32	0.503	0.051	1.24	5.9	11.8
190	0.159	0.315	0.478	0.029	1.31	5.9	12.3

LL- lower limit of plant extractable soil water, DUL- field capacity, SAT- saturated water content, CEC- cation exchange capacity

Table S2. Soil properties of Amnura series input for APSIM model

Layer Depth (cm)	KS (mm/day)	BD (g/cc)	Airdry (mm/mm)	LL15 (mm/mm)	DUL (mm/mm)	SAT (mm/mm)
0-10	10.000	1.210	0.114	0.118	0.289	0.511
10-15	5.000	1.270	0.182	0.105	0.261	0.491
15-25	24.000	1.300	0.228	0.141	0.293	0.482
25-45	24.000	1.270	0.228	0.127	0.283	0.493

KS- hydraulic conductivity of the soil at saturation

Table S3. Weather data input parameter for APSIM and DSSAT models

Date	Solar radiation	Max daily temperature	Min daily temperature	Precipitation	Dewpoint temperature	Average wind speed	Vapor pressure	Relative humidity
yyyy-mm-dd	MJ/m2/d	(°C)	(°C)	mm	(°C)	m/s	hPa	%
W_DATE	SRAD	TMAX	TMIN	RAIN	TDEW	WIND	VPRSD	RHUMD
2018-07-01	13.8	25.4	12.6	0	10.1	0.5	12.4	36
2018-07-02	8.3	25.4	12.7	0	12.8	0.4	14.8	43
2018-07-03	10.4	25.3	12.6	8.6	10.8	0.5	13	38
2018-07-04	16.1	24.6	12.8	0	9.8	1.2	12.2	37
2018-07-05	15	25.4	12.6	0	8.8	1.7	11.4	33
2018-07-06	15.7	25.5	12.6	0	7.1	1.5	10.1	29
2018-07-07	16.3	25.6	12.7	0	8.1	1.6	10.8	31
2018-07-08	16.1	25.9	12.9	0	11.4	1.4	13.5	38
2018-07-09	16.1	25.3	13.1	0	10.4	1.2	12.7	37
2018-07-10	14.6	25.8	12.7	0	12.1	1.4	14.1	40

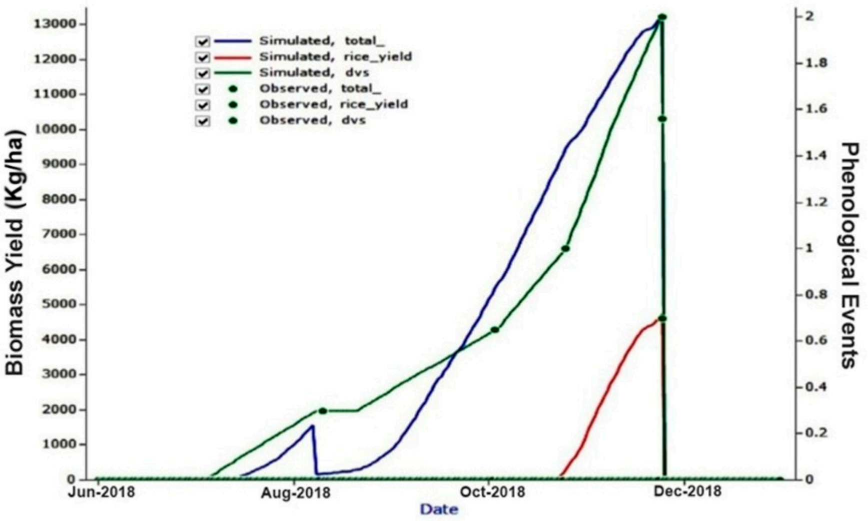

Figure S1. Calibration and validation output (observed and simulated) for the Rice cultivar (BR11- T. Aman) using APSIM-Rice model.

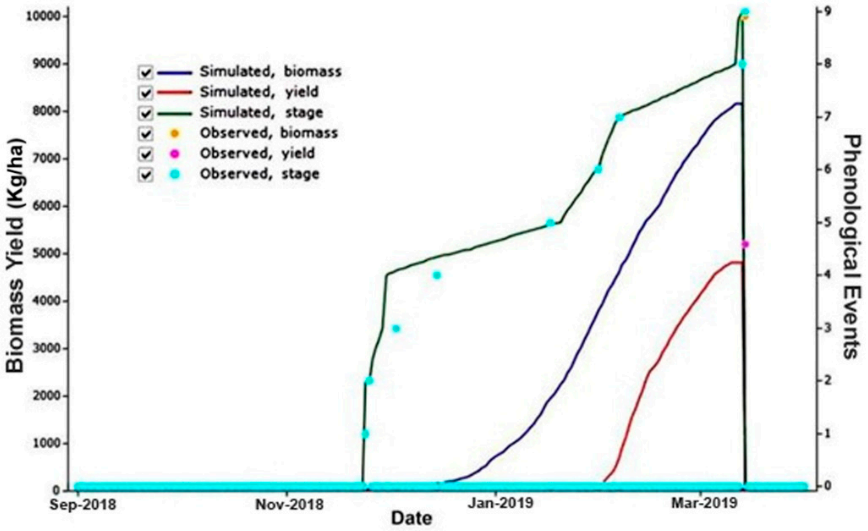

Figure S2. Calibration and validation output (observed and simulated) for the wheat cultivar Shatabdi using APSIM-Wheat model.
